# A turning point after FACT: a qualitative study of family members’ experiences and follow-up provided by flexible assertive community treatment

**DOI:** 10.1186/s12913-025-13485-z

**Published:** 2025-09-30

**Authors:** Randi Martinsen, Anne Signe Landheim, Berit Arnesveen Bronken

**Affiliations:** 1 Faculty of Social and Health Sciences, Department of Health and Nursing Sciences, University of Inland Norway, Elverum, Norway; 2 Norway and Centre for Co-Occuring Substance use Disorders and Mental Illness, Innland Hospital Trust, Hamar, Norway

**Keywords:** Experiences, FACT, Family members, Family carers, Flexible assertive community treatment, Focus groups, Relatives, Severe mental illness, Qualitative

## Abstract

**Background:**

Service users living with severe mental illness are often in need of treatment from different health care professionals. Their family members play an important but underreported role in their treatment and care. Following the implementation of the flexible assertive community treatment (FACT) model in Norway since 2013, FACT teams have been evaluated from different perspectives. The overall aim of this study was to explore how family members of people living with SMI experience their situation and the follow-up provided by FACT teams.

**Methods:**

Forty-one family members aged 31 to 78 years took part in nine focus groups representing seven FACT teams from both rural and urban areas in Norway. Most of the participants had parental roles. The data was analysed using qualitative content analysis.

**Results:**

The results revealed the following two main themes and five sub-themes: (1) An all-consuming and demanding role (*A life of love*,* care and despair* and *A life affecting health and well-being)*, and (2) A turning point after FACT (*From a patchwork to more integration and continuity*, *Family involvement as a vital part of care* and *Availability of support outside regular opening hours).*

**Conclusions:**

Being family members of service users with SMI are experienced as an all-consuming and demanding role. The family members experience the FACT team as an important support in their day-to-day life. However, they wish to receive more support and inclusion in the treatment than they currently do. Further strengthening the involvement and collaboration between the family members and the FACT teams is recommended. Psychoeducational interventions arranged by the FACT team could support family members’ situations. To share their stories with professionals and/or peers would further contribute to their well-being. Extending the opening hours of FACT is recommended as it might mitigate family members’ everyday challenges and improve their subjective well-being.

**Supplementary Information:**

The online version contains supplementary material available at 10.1186/s12913-025-13485-z.

## Background

Service users living with severe mental illness (SMI) and complex problems need interdisciplinary treatment and cross-sectoral support. In most cases, family members play an important but underreported role in the treatment and care of these service users. “Family member” is understood in Norway as not limited to relatives by blood or marriage but can include e.g. close friends. Being a family member of these service users is described as being in a challenging situation, struggling with ongoing worries and anxiety, and having to deal with demanding situations [1, 2]. This article focuses on family members’ experiences of their situation and the follow-up provided by flexible assertive community teams (FACT).

Living with service users with mental illness and complex problems might make family members feel stigmatized [3, 4] and discriminated [3]. Family bonds affect the meaning of everyday living in this situation, where life is unpredictable around the clock and there are constant barriers impacting one’s own life [2] and that of the entire family [1, 5]. Acting in the wrong way can worsen the state of the service users and the relationship to family members and make it more difficult for the latter to communicate honestly and to diminish control. Family members have described the difficulty of being open about the service users’ condition and explained how they are occupied with thoughts of protecting others, especially the children in the family [1].

Family involvement in care provision is reported to be problematic for the service user, the mental health care professionals and the family members themselves [6]. Nevertheless, due to their challenging life, family members wish to be involved and included in treatment and care. Negative judgements such as being troublemakers, pushy or demanding have been identified [2]. Several unmet needs have been reported, such as being respected, valued, taken seriously and emotionally supported by professionals, as well as being informed both formally and informally [1, 2, 6, 7]. Despite having adverse experiences, family members report a need to be involved in the treatment and to receive support as relatives from mental health services [1, 7].

To meet the needs of service users with SMI, the FACT model, developed in the Netherlands [8, 9], was established in Norway in 2013 [10, 11]. In this model, treatment and support are flexible and outreaching, being offered by a local team to support service users where they currently live. An overall goal of the FACT model is to strengthen and coordinate health care services offered to persons with SMI who need treatment and support from different levels of care. Furthermore, the FACT model aims to improve service users’ psychosocial functioning and enable them to live a better life in the community [12]. This implies that an overall goal is to provide integrated and coordinated care by being attentive and accessible [8, 9].

FACT teams are multidisciplinary and recovery-oriented, often including a nurse, a psychiatrist, a psychologist, a peer specialist, a social worker, an addiction specialist, a peer recovery support specialist and a supported employment specialist. Moreover, all service users have their own case manager, i.e. a primary contact responsible for the coordination of the professionals involved in the care and treatment [8]. The care provided depends on individual needs and thus varies between intensive and less intensive support, for shorter or longer periods. This way of organizing health care might ensure continuity in the support system and be tailored to the service users’ specific needs [10]. According to the Norwegian version of the FACT manual, service users should have access to health care services around the clock either directly from the FACT team or in close collaboration with other relevant services. However, most of the FACT teams in Norway do not have opening hours in the evening, at night or at weekends [11]. The holistic and recovery-oriented basis of the FACT model means that the FACT team is also responsible for supporting family members in their roles by offering advice, psychoeducation and guidance for contact with peer support groups [9, 11]. FACT teams in Norway are mostly organized as a binding collaboration between the municipality and specialist mental health services. The FACT teams are supposed to provide nearly all services from the team [11].

Research shows that FACT teams have a positive impact on service users’ quality of life [13], psychosocial functioning [14] and participation in daily activities [15]. Receiving FACT has also been expressed as preventing coercion [16]. The number of hospital admissions has decreased [13, 17, 18] and the number of service users in remission has increased during a two-year period [13]. Results from a randomised controlled trial in Germany based on the principles of the FACT model, found that participants diagnosed with mental disorders followed up by multiprofessional teams improved significantly on perceived empowerment, subjective quality of life and satisfaction with mental health services compared to the control group receiving treatment as usual. However, the authors found no differences on the severity of mental illness between the groups [19].

Research focusing on family members’ experiences and involvement with FACT teams is limited. However, a study found that receiving care and support from assertive community teams (ACT) was felt to be superior to previous collaboration with mental health care professionals, with family members highlighting openness and collaboration as essential [20]. Including family members in the therapy provided to service users has been mentioned as positive [21]. Providing therapy to family members has been found to be challenging but necessary from the perspective of heath care professionals [8, 22]. Barriers to the involvement of family members might be individual stressors, relationships within the family and organizational challenges [22]. Legal and ethical issues such as lack of consent and duty of confidentiality are also emphasized as barriers to involvement [22, 23]. Involvement of family members of persons with psychotic disorders has been found to be arbitrary. However, a focus on knowledge development of health care professionals was found to improve their skill in involving family members [23].

Following the establishment of FACT teams in Norway, the teams have been evaluated from different perspectives. This sub-study explores family members’ experiences of their situation and the follow-up and support provided by their local FACT team.

### Aim

The overall aim of this study was to explore how family members of people living with SMI experience their situation and the follow-up provided by FACT teams.

The following research questions were formulated:

- How do family members describe their situation living with a service user with SMI?
- How do family members experience the follow-up they receive by FACT teams?
- how do family members experience the collaboration with FACT teams?

## Methods

### Design

The study has an exploratory, descriptive design, using a qualitative method involving purposive sampling.

### Setting and recruitment

Participants were recruited from seven FACT teams in rural and urban areas across Norway. To be included in the study, participants had to be family members of individuals living with SMI, be 18 years of age or over and receiving treatment and care from a FACT team for at least one year. Moreover, participants had to have regular contact with the service user.

Recruitment of participants for this study was conducted in multiple stages. The project leader of the research team (ASL) initiated contact with the managers of each FACT team. These managers provided both oral and written information about the study to all their service users of the FACT team and obtained consent to contact their family members for participation in focus groups. Once written consent was secured from the service users, the family members of those who gave consent were subsequently contacted. The family members received oral and written information and written consent was obtained from the family members who agreed to participate in the focus group interviews (ref. Declaration section).

### Data collection

Focus group discussions were considered to provide rich data through interaction, experience sharing and discussions among participants in similar situations and roles [24, p. 515]. The authors developed a thematic interview guide based on the principles of the FACT model [11] and existing literature about family members’ experiences [1–5] to facilitate the discussions regarding their situation living with a service user with SMI, the follow-up they receive by FACT teams and the collaboration with FACT teams (See supplementary file). The thematic interview guide was supplemented with additional detailed questions based on the discussions and interactions in each focus group.

Nine focus group discussions were conducted between June 2019 and May 2023. The number of participants in each focus group ranged from three to seven. Two of the authors (RM and BAB) facilitated and conducted the focus groups and alternated as both moderator and co-moderator. The moderator mainly organized the conversation, and the co-moderator assisted the moderator, ensured that all themes in the thematic guide were discussed among the participants, took notes during the sessions and summarized the content of the focus group discussions at the end.

### Characteristics of the participants

A total of 41 family members consented to take part in the study. The characteristics of the participants are presented in Table 1.

**Table 1:** Characteristics of the participants

	*n* = 41
**Age**	
*Mean (range)*	59 (31–78)
**Gender**	
*Female*	29
*Male*	12
**Relationship**	
*Parent*	29
*Sibling*	4
*Partner*	2
*Child*	3
*Other*	3

None of the participants knew each other before study participation.

### Analysis

The focus groups were audio recorded and transcribed verbatim. The transcripts were then analysed using qualitative content analysis, as this method is useful for focusing on manifest and latent content regarding both subject and context [25–27].

In the initial step of the analysis, all the transcripts were read and reread several times, to obtain a sense of the whole. Next, the research group searched for meaning units that illuminated specific experiences related to the research questions. The meaning units were then coded and condensed into five sub-themes, abstracted into the following two main themes: *An all-consuming and demanding role* and *A turning point after FACT*. An overview of the themes is presented in Table 2.

**Table 2:** Overview of main themes and sub-themes

Main theme: **An all-consuming and demanding role**
Sub themes:
*A life of love*,* care and despair*
*A life affecting health and well-being*
Main theme: **A turning point after FACT**
Sub themes:
*From a patchwork to more integration and continuity*
*Family involvement as a vital part of care*
*Availability of support outside regular opening hours*

## Results

### An all-consuming and demanding role

This theme presents how the family members experienced their role and everyday life, revealed in the following sub-themes: *A life of love*,* care and despair* and *A life affecting health and well-being.*

#### A life of love, care and despair

All the participants demonstrated a great need to tell their stories about their situation and role as family members both before and after the establishment of FACT. Many had several decades of experience of being responsible for all practical and emotional situations, being a carer around the clock. They described their situation and a life with mixed feelings of love, grief, anxiety, shame and distress. Although the distress and responsibilities were considerable, their fundamental love and care for their family member coloured their stories. All highlighted the importance of never giving up, especially not giving up on their own child: “*We’re the only ones he* [the adult child] *has”.*

Many described their anxiety about what could happen next; they feared their loved ones’ lives were at risk. They had to be constantly ready to face all kinds of situations. Living under mental pressure for several years had increased their level of chronic stress. One mother said that every time the doorbell rang, she was very frightened: *“My body reacted as if it was the priest. I’m quite simply afraid that he* [her son] *will die!”*

The child’s reaching the age of majority marked a turning point for some of the parents. They found that they lost all their rights to information and involvement in the recovery process. At the same time, some expected to continue to take care of and be responsible for an adult child. One mother said: *“We have no rights even if he’s suicidal and needs protection from us* [parents] *around the clock.”*

The entire family’s life was affected and they all needed help and support. Most tried to protect themselves and their families from stigma, as expressed by one of the siblings: “*These patients are at the lowest level of the social ladder of all patients. That’s definitely what I feel”.*

Caring for a severely ill family member greatly challenged their day-to-day living, relations and roles. They described their own situation as living in an all-consuming role, needing to be alert and in emergency preparedness around the clock, as described by a sister and her parents: *“We visit his home many times a day*,* he calls us*,* he loses his belongings. It’s a never-ending mess!”*

The family members were concerned about the lack of meaningful activities for the service users. They felt responsible for daily activities, and it hurt them to think that the service user was sitting alone with nothing to do every day. Meaningful activities were seen as essential and could have alleviated the situation for both service users and family members: “[They need more] *daily activities. That would be a great help. On their own terms”.* The family members therefore suggested that the FACT teams should be more attentive to motivating service users to participate in meaningful activities such as sports, outdoor life, courses, clubs, returning to work or having a small job. Several called for tailored activity schedules. Others told positive stories of involvement in meaningful activities such as going to a café or music therapy.

The relationships and interplay between all the family members were challenged and it was difficult to solve the problem. Too much attention was given to the ill family member. One mother referred to her daughter’s frustration: *“Mum*,* you must remember that you have two more children as well!”* Another mother said that she could not visit her daughter who lived away from home because it was too risky to leave the ill family member alone at home. Her daughter had expressed disappointment and grief. Although the situation had changed and had improved greatly for some since FACT started, it was still difficult to live a “normal” family life as all their energy was spent on their unpredictable situation.

The FACT teams met the family members’ various needs in different ways. Indirectly, any support that enhanced the ill person’s care and safety was of benefit to all the family. One partner talked about her difficult ethical dilemma in deciding to move out and leave her ill partner alone to care for their four children. She had not been offered any help from the FACT team and was very frustrated, angry and disappointed.

*I think they* [the FACT team] *have been very absent. He* [her partner] *returned home following treatment more than six months ago. I had to make contact myself. I feel fussy and insistent*,* and they don’t understand. That’s very frustrating* […] *It’s vital that they listen to us.*

The participants expressed a great need for knowledge and advice on how to communicate and handle difficult situations and reactions. Not knowing how to respond to comments was a dilemma. One of the daughters said: “*I need some more education.* […] *How am I supposed to answer when my Dad’s telling me that he’s planning to commit suicide?”*

#### A life affecting health and well-being

The family members’ role affected their health and well-being. Several of the participants were on partial or complete sick leave and others received disability benefits after years of psychosocial distress. Taking care of adult home-dwelling children left some of the parents feeling that they were stuck in their homes, which placed them in various dilemmas. Although they wanted to take care of and protect the person suffering from SMI, they expressed considerable frustration at being in a situation where they were unable to live their lives as they wanted. Some of the people with SMI acted as the head of the family and controlled the family activities. One mother said: “*He* [her son] *is controlling everything. So we’re shut in here and* [totally] *tied up”.*

The father of a son who had made several suicide attempts stated:

*I’ve been depressed and more isolated. I used to be a very extroverted person taking part in many different activities. Now I cross the road to avoid meeting people. I think it’s a great pity. This isn’t the way I used to be. There aren’t many happy days now.*

Others mentioned frightening events which left them alone with great responsibility. One mother said:

*Once he* [her son] *locked me in the bathroom* […] *said I was a paedophile and ought to be behind bars and in jail. I was terrified. I’m afraid to be alone with him – that’s terrible for a mother. He’s very sick.*

However, some found it helpful to go to work; one of the wives said: *“To me*,* going to work is a breathing space”*. Some who were working found it difficult to include colleagues in their private situations. However, they highlighted the importance of informing their superior about their situation in order to be understood. Employers’ willingness to provide flexibility was seen as a prerequisite to cope with working outside the home. Some explained that they were unsure of how their future would be, and whether they would be able to return to their job in the future. One of the mothers said: “*I don’t think I’ll be able to go back to work. I feel completely down”.*

However, although they felt that their home was like a prison, some carers managed to put their responsibility on hold for periods. Making such decisions was not easy but seen as necessary for their well-being and to endure life. One father, speaking on behalf of himself and his wife during one of the focus groups, said: *“We must live our own lives. We’re going on holiday for weeks. We can’t give support 24/7. That would kill us.”* He and his wife pointed out that they needed support and verification from the FACT team to prevent them from feeling guilty about taking some time off and looking after themselves.

### A turning point after FACT

All participants in the focus groups stated that implementation of the FACT model represented a change in care that needed to become a permanent part of the organization of mental health care. However, there were still some challenges that complicated everyday life for themselves and the service users. This theme is expressed by the following three sub-themes: *From a patchwork to more integration and continuity*, *Family involvement as a vital part of care* and *Availability of support outside regular opening hours.*

#### From a patchwork to more integration and continuity

The FACT teams represented a positive and welcome turning point for all participants in the current study. Prior to the establishment of local FACT teams, the health care service system was described as fragmented and random, as an adult child explained:

*We used to feel that treatment was a kind of patchwork of random options that the GP came up with (…)*,* literally kind of here*,* there and everywhere. (…) It gets to be very stressful when you have to repeat the same story over and over again to new people (…) This is the first time we as family members have felt that he* [the service user] *has received systematic care.*

The case manager in the FACT team played an essential role and the participants mostly felt that collaboration with the team was valuable. Having someone to share the responsibility and to call and discuss situations with was emphasized as very important, as explained by a mother: *“FACT has relieved a lot of responsibility from Mum’s shoulders”.* The participants felt that they were part of the collaboration and were considered, listened to and trusted. One of the mothers explained how she felt about the contribution of the FACT team:

*I’m not missing anything because it’s changed so much for the better. I’ve been used to fighting all the time*,* but suddenly there’s actually someone who takes hold of the situation*,* takes action and it all happens very quickly.*

Availability of support was now easier and smoother, highlighting that the FACT team was attentive and supportive. Most of the participants felt that they were listened to and taken seriously. Knowing whom to call and being able to make contact was reassuring.

The relationship between the family members and the case manager was regarded as essential. However, this relationship differed somewhat between FACT teams. Personal and professional competence and attitude were emphasized as crucial, but depended on the individuals working in the FACT team, as expressed by a mother:

*It all depends on the people you meet. They* [the case managers] *relate to* [the service user] *in a careful way and you get to trust them completely. He* [her son] *actually answers the phone when they call*,* which is different from what he usually does.*

Some told positive stories about how the various needs of the families were met. A mother who had been a single parent for many years with three children, one of whom was seriously ill, had called for family support. After her ill son received FACT, his healthy but emotionally affected brother received priceless support from the FACT team. This mother emphasized that the need for family support was most prominent in the beginning when the situation was new and unfamiliar.

#### Family involvement as a vital part of care

Although the family members were pleased with the treatment and care provided by the FACT team and agreed that it had brought many valuable changes to mental health care, there were still several areas that could further alleviate their life situation. Several felt that communication between the family members and the FACT team was one-way communication. The family members wished that the case manager would initiate contact and invite them to more systematic collaboration.

Their role as family members involved with the FACT team was described as essential and important, but complicated and challenging: *“Without us* [parents] *he* [the son] *wouldn’t have been alive – we’re a vital part of his care – without any rights”.* They wanted to be more involved and informed without being left aside. They felt uncertain about how to communicate and act with the service users without making the situation worse. They expressed a desire for more knowledge, guidance and systematic support from the FACT team, as well as a clearer delineation of mutual roles, responsibilities and expectations. Several of the family members said that the focus group discussion during the current study was the first time they had ever shared their experiences with others in the same situation, which they found valuable. Some of the family members had occasionally been offered talks and guidance from the case manager of their FACT team, which they had found very useful and welcome. One of the mothers with a daughter living with SMI for more than 20 years explained:

*I was invited to talk to two mental health nurses because we* [she and her husband] *had never talked to anyone.* […] *It was very nice because I could tell them about everything from the beginning. It’s actually the first time I could speak openly. That was very satisfying.* […] *Now we’re more relaxed.*

A safe place to live was also a big concern and was expressed as a basic condition for a worthwhile life. Living alone required complex skills to fulfil social obligations. One of the siblings emphasized the patients’ needs for support to manage their lives: *“They need help to live!”* Help with cleaning and shopping from a dedicated person might have benefitted both the service user and the family members.

#### Availability of support outside regular opening hours

A recurring discussion in all focus groups concerned the availability of help and support in difficult situations occurring outside the FACT services’ regular opening hours, which were from 8 am to 4 pm on weekdays. Outside these hours, availability of help was described as almost “*absent”* or *“complicated”*. The family members were concerned about acute situations with exacerbation of symptoms or other critical situations, which often occurred in evenings, nights, weekends and holidays. One of the mothers expressed it in this way:

*It’s very often at weekends or holidays things happen. … It was when they* [the FACT team] *took a holiday everything happened*,* Easter*,* Whitsun and things like that*,* so when you really needed them* [FACT team] *to be staffed*,* I had to sit there on call and be the staff they* [FACT] *should have been.*

Availability of members of the FACT team around the clock would have benefitted both service users and family members: *“Simply being able to call someone who knows the person’s situation would really make things easier”.*

Staffing and access to professional resources differed between municipalities, especially in rural areas. Because of turnover among FACT team members or members who worked part-time or were on sick leave, it was difficult for several of the family members to be certain that the service users would receive care in accordance with their needs. Several also questioned doctors’ knowledge of the needs and challenges of the service user, which often led to inappropriate treatment and solutions, as expressed by one of the mothers:

*It’s always been a battle. A general practitioner that doesn’t understand and only meets the patient for the ten to twenty minutes when we’re there and just throws the ball back* [to us].

Collaboration between the various services within the municipality and across service levels was considered to hamper continuity and integrated care. To contact the emergency ward was often frustrating and complicated, involving an outright refusal, a long waiting time, and a lack of understanding of the situation, as one of the mothers expressed:

*We have no places to go. They say we can call the emergency services*,* and they’ll call us the next day. Then I’ve tried to call the ambulance*,* and when they hear about mental health problems*,* they just ask if it’s urgent to save the patient’s life.* […] *No collaboration. Every professional want to play it safe and handle the situation according to their own ideas.*

Moreover, legislation such as confidentiality requirements and the need for voluntary admission and consent were perceived by many as obstacles to treatment, care and continuity. The family members referred to situations when their loved ones had psychotic and/or progressive symptoms of a developing manic crisis and demonstrated a low degree of self-insight, which was difficult to understand for doctors who did not know the individual’s illness history. They often found that the service users were not considered ill enough for admission to hospital, which meant that they ended up at home with a seriously ill person they lacked competence to handle. Such experiences were even more traumatic if the police were involved, and it was necessary to use force and coercion to gain control of a situation. One partner said (crying): *“When acute problems occur*,* when you need help*,* in fact you have to get the police at your door.* […] *He’s not a criminal; he’s just very ill.”*

On the other hand, to consent to compulsory admission and treatment in specialized hospital units was also traumatic, as one daughter said:

Being in such situations as a relative and fighting for compulsory admission, that’s incredibly tough. Standing up for what you do, and then the feelings you’re left with! It hasn’t happened since he started with the FACT team.

Access to support around the clock would probably have prevented unnecessary crises and admissions to hospital.

## Discussion

The results of this study revealed that the family members of service users with SMI take on an all-consuming role and live a challenging life of love, care and despair filled with various dilemmas. The participants stated that receiving care from FACT teams was a positive turning point that relieved them of some responsibility as family members and ensured care and support for themselves and the service users. In the following, the results are discussed in detail.

### Family members – an essential and burdensome role

All participants in this study had several years’ experience as family members of service users living with SMI and complex problems. Most of the participants were mothers or fathers, and their freely told stories in the focus groups emphasized that a parental role is a lifelong role of living with concerns for one’s children. This study emphasizes that family members make a positive and important, but more or less invisible, contribution to mental health care in Norway. Family members living with service users suffering from SMI live a life filled with stress and contribute substantially to health care services in line with government recommendations [28]. Nevertheless, their contribution in society needs to be made visible and the family members themselves are also in need of care and support, as they are at risk of developing both physical and mental illnesses [29].

Some of the participants in this study had the opportunity to tell their stories to the case managers in the FACT team, while others missed being able to tell their story to a professional health care worker. Being a family member of a person with SMI is emphasized as a complex role [1, 2, 23, 30] and described as a burden that might lead to illness both within and outside the context of mental health care [1, 2, 29, 31]. The experience of a burden is individual, ranging from a societal burden to struggling with personal thoughts such as ambivalence, guilt and responsibility [31].

The stories told by the participants in this study emphasize that people living with mental distress often need to tell their illness stories [32]. According to narrative psychology, narratives play an existential role in the process of constructing the meaning of events and experiences through language and interaction with others and society [33, 34]. The results of the present study show that the ability of the participants to tell their story to a member of the FACT team could increase their subjective well-being. This emphasizes that opportunities to discuss the situation should be included as a part of the psychoeducational support provided to family members to fulfil the aim of the FACT guidelines in line with the holistic and recovery-oriented basis of the FACT model [9, 11].

Participating in focus groups enabled the participants to tell their stories in a group of peers. Although they expressed some tension or anxiety before meeting unknown people in the focus groups, the opportunity to tell one’s story in the groups was welcomed and a new and positive experience. This indicates that telling stories in a group of peers and professionals could ease the burden of being a family member. This is in line with the aim of the FACT model, which also suggests regular discussions within families [9, 11].

The results showed that the participants missed having knowledge of the illness trajectory, as seen in other studies revealing that families need information and knowledge to enable them to support their chronically ill family member [30]. Discussions in peer groups of family members focusing on everyday experiences and education about the psychological mechanism of the SMI might give family members some tools to make for smoother management of day-to-day living. Arranging groups of family members could even be a positive economic contribution to developing a sustainable healthcare system in the future. However, systematic individual or group-based psychoeducation is necessary to improve family members’ knowledge and skills to handle communication and act in difficult situations. This could promote the health and well-being of both family members and service users.

Many of the service users had few or no activities during the week to support their recovery process, which the family members viewed as an area for improvement. Knowing that the service user was able to participate in a meaningful activity could relieve the family members of troublesome thoughts about how to encourage the service users to take part in society in a meaningful way.

Although the FACT teams represented a positive turning point and reduced the everyday burden of the participants in the current study, the lack of access to adequate support from members of the FACT teams outside regular opening hours still gave service users and family members practical and psychological problems and distress, particularly in crises and acute situations. This has also been reported as a barrier in a study of service providers’ experiences of collaboration with FACT teams in Norway three years after the FACT model was introduced. Those findings also document a positive change in terms of bridging some gaps in the existing system, but the FACT teams’ working hours represented a barrier to both professionals in primary and secondary care. They suggested that 24-hour availability of FACT would have provided more competent staff in crises [10]. The original FACT model from the Netherlands recommends services around the clock [9].

### Strengths and limitations of the study

To the best of our knowledge, this is the first study focusing on the family members of service users receiving care from FACT teams in Norway, thus representing a valuable aspect of the care of service users living with SMI. The results of this study concerning the family members’ experiences and involvement with FACT teams will add to the body of knowledge in the field and thus help to further develop FACT teams for service users and their family members. Nevertheless, more research is needed to enhance knowledge of the important and demanding role of family members.

This study was conducted in a Norwegian cultural context. Including other contexts could have shown different results, as the legislation and regulation of healthcare systems differ between countries. However, there is reason to believe that individual experiences of living in families that include severely ill people may be comparable in other similar contexts.

The purposive sampling method might have included relatively well-educated and resourceful family members. A limitation is therefore that the study did not include family members with their own mental health problems or service users themselves. Involving those groups in the study could have yielded different results.

## Conclusion

Being family members of service users with SMI are experienced as an all-consuming and demanding role. The family members experience the FACT team as an important support in their day-to-day life. However, they wish to receive more support and inclusion in the treatment than they currently do.

Further strengthening the involvement and collaboration between the family members and the FACT teams is recommended. Psychoeducational interventions arranged by the FACT team could support family members’ situations. To share their stories with professionals and/or peers would further contribute to their well-being.

Extending the opening hours of FACT is recommended as it might mitigate family members’ everyday challenges and improve their subjective well-being.

## Supplementary Information

Below is the link to the electronic supplementary material.

Supplementary Material 1

## Data Availability

The datasets generated and analysed during the current study are not publicly available due to the confidentiality of the participants.

## Abbreviations

ACT: Assertive community treatment
FACT: Flexible assertive community treatment
SMI: Severe mental illness
