# Supplementary material for: A turning point after FACT: a qualitative study of family members’ experiences and follow-up provided by flexible assertive community treatment
Source: BMC Health Serv Res. 2025 Sep 30;25:1254. doi: 10.1186/s12913-025-13485-z (PMC12482409; doi:10.1186/s12913-025-13485-z)
Supplement: Supplementary file 1 — Supplementary Material 1 [file 12913_2025_13485_MOESM1_ESM.docx]

Appendix

**Thematic interview guide**

Introduction

Welcome and presentation of the project, the moderator and co-moderator.

We will mainly focus the discussion on family caregivers’ experiences and situation, and we are interested in all your experiences – positive and negative (if any). We will encourage you to keep the discussion in the group by yourself and not talk with others about the conversation in the group.

The aim:

- to explore family members’ experiences with the follow-up provided by FACT teams.

You are secured confidentiality.

The timeframe: one to two hours (including a break in between).

Short presentation of the participants around the table

- How do family members describe their situation living with a service user with SMI?
- How do family members experience the follow-up they receive by FACT teams?
- How do family members experience the collaboration with FACT teams?

Theme 1

Please tell us about your experiences of being a caregiver to a family member with SMI followed-up by the FACT team. *Examples are welcomed.*

Theme 2

Please tell us about your experiences of the follow-up by the members of the FACT team

Theme 3

Please tell us about your experiences of your collaboration with the FACT team

To conclude:

What are the positive and less positive elements in the follow-up by the FACT team?

Do you have any suggestions to improve the quality of the follow-up?

Do you have other suggestions or anything else you want to supplement?

*Thank you for your participation! Your experiences and suggestions are important for increase the quality of the follow-up by FACT teams.*
